# PReS-FINAL-2070: Efficacy of biologic treatments in juvenile idiopathic arthritis with a polyarticular course: an indirect comparison

**DOI:** 10.1186/1546-0096-11-S2-P82

**Published:** 2013-12-05

**Authors:** L Sawyer, A Diamantopoulos, HI Brunner, F De Benedetti, N Ruperto, F Dejonckheere, C Keane

**Affiliations:** 1 Symmetron Limited, London, UK; 2 PRCSG, Cincinnati, USA; 3 IRCCS Ospedale Ped Bambino Gesú, Rome, Italy; 4 PRINTO, Genoa, Italy; 5 F. Hoffmann-La Roche, Basel, Switzerland; 6 Roche, Welwyn, UK

## Introduction

To date there are no head-to-head trials comparing the efficacy of biologic treatments for polyarticular-course JIA (pcJIA).

## Objectives

To use statistical methods to estimate the relative efficacy of biologic treatments, alone and in combination with methotrexate (MTX), in the management of pcJIA by means of indirect comparison of randomised controlled trials (RCTs).

## Methods

Based on a literature review, we identified RCTs of abatacept, adalimumab (ADA), etanercept, infliximab and tocilizumab (TCZ) in pcJIA. Comparative effectiveness was estimated on the reported American College of Rheumatology response rates (JIA ACR30/50/70/90) measured at the end of the randomised, double-blind phase by means of a Bayesian indirect comparison using a fixed-effects ordered probit model. Probabilities of achieving different levels of JIA ACR response were calculated for biologic treatments and placebo using all observed comparisons.

## Results

The 5 RCTs identified showed differences in reporting JIA ACR responses with regard to methods of non-responder imputation during the blinded, controlled phase, allowing only for the comparison of ADA and TCZ. In the base-case analysis, for a JIA ACR30 placebo response of 31%, TCZ monotherapy had a higher predicted probability of JIA ACR30 (62%), JIA ACR50 (59%), JIA ACR70 (54%) and JIA ACR90 (35%) response than ADA monotherapy (53%, 49%, 44% and 26%, respectively). On MTX background therapy and a JIA ACR30 placebo response of 53%, ADA had a higher expected probability of response at JIA ACR30 (76%), JIA ACR50 (75%), JIA ACR70 (66%) and JIA ACR90 (49%) than TCZ (72%, 70%, 61% and 44%, respectively). In neither monotherapy nor combination therapy did differences between TCZ and ADA reach statistical significance. Differences in the study populations, including previous use of biologics, were explored with sensitivity analysis.

## Conclusion

Based on JIA ACR response rates from this analysis, the expected efficacy of ADA vs TCZ appears comparable in pcJIA. These data should be interpreted in the context of differences in the duration of the withdrawal phase, which was shorter in the TCZ study (CHERISH) than in the ADA trial and might have resulted in a smaller difference in the number of flares observed between placebo and TCZ. Differences in previous exposure to biologics might also have affected the results.

## Disclosure of interest

L. Sawyer Consultant for: F. Hoffmann-La Roche, A. Diamantopoulos Consultant for: F. Hoffmann-La Roche, H. I. Brunner Consultant for: Novartis, Genentech, MedImmune, EMD Serono, AMS, Pfizer, UCB, Janssen, Speakers Bureau: Genentech, F. De Benedetti Grant/Research Support from: Abbott, Pfizer, BMS, Roche, Novimmune, Novartis, SOBI, N. Ruperto Grant/Research Support from: Abbott, AstraZeneca, BMS, Centocor, Lilly, Francesco Angelini, GSK, Italfarmaco, MerckSerono, Novartis, Pfizer, Regeneron, Roche, Sanofi Aventis, Schwarz Biosciences, Xoma, Wyeth, Consultant for: Abbott, AstraZeneca, BMS, Centocor, Lilly, Francesco Angelini, GSK, Italfarmaco, MerckSerono, Novartis, Pfizer, Regeneron, Roche, Sanofi Aventis, Schwarz Biosciences, Xoma, Wyeth, Speakers Bureau: Abbott, Boehringer, BMS, Novartis, Astellas, Italfarmaco, MedImmune, Pfizer, Roche, F. Dejonckheere Employee of: F. Hoffmann-La Roche, C. Keane Employee of: Roche.

